# Self-Organization Regimes Induced by Ultrafast Laser on Surfaces in the Tens of Nanometer Scales

**DOI:** 10.3390/nano11041020

**Published:** 2021-04-16

**Authors:** Anthony Nakhoul, Claire Maurice, Marion Agoyan, Anton Rudenko, Florence Garrelie, Florent Pigeon, Jean-Philippe Colombier

**Affiliations:** 1 UJM-St-Etienne, CNRS, Laboratoire Hubert Curien UMR 5516, Institute of Optics Graduate School, Univ Lyon, F-42023 Saint-Etienne, France; anthony.nakhoul@univ-st-etienne.fr (A.N.); marion.agoyan@univ-st-etienne.fr (M.A.); garrelie@univ-st-etienne.fr (F.G.); florent.pigeon@univ-st-etienne.fr (F.P.); 2 Mines Saint-Etienne, CNRS, Univ Lyon, UMR 5307 LGF, Centre SMS, F-42023 Saint-Etienne, France; maurice@emse.fr; 3 Arizona Center for Mathematical Sciences and College of Optical Sciences, University of Arizona, Tucson, AZ 85721, USA; antmipt@gmail.com

**Keywords:** ultrafast laser nanostructuring, self-organization, nanobumps, femtosecond laser, LIPSS, nanopatterns

## Abstract

A laser-irradiated surface is the paradigm of a self-organizing system, as coherent, aligned, chaotic, and complex patterns emerge at the microscale and even the nanoscale. A spectacular manifestation of dissipative structures consists of different types of randomly and periodically distributed nanostructures that arise from a homogeneous metal surface. The noninstantaneous response of the material reorganizes local surface topography down to tens of nanometers scale modifying long-range surface morphology on the impact scale. Under ultrafast laser irradiation with a regulated energy dose, the formation of nanopeaks, nanobumps, nanohumps and nanocavities patterns with 20–80 nm transverse size unit and up to 100 nm height are reported. We show that the use of crossed-polarized double laser pulse adds an extra dimension to the nanostructuring process as laser energy dose and multi-pulse feedback tune the energy gradient distribution, crossing critical values for surface self-organization regimes. The tiny dimensions of complex patterns are defined by the competition between the evolution of transient liquid structures generated in a cavitation process and the rapid resolidification of the surface region. Strongly influencing the light coupling, we reveal that initial surface roughness and type of roughness both play a crucial role in controlling the transient emergence of nanostructures during laser irradiation.

## 1. Introduction

Femtosecond laser sources allow us to explore the material’s behavior under swift photoexcitation. This represents a privileged way to bring material surfaces in extreme states of temperature and pressure far from equilibrium. During the relaxation stage, out-of-equilibrium systems usually exhibit pattern formation as a result of spontaneous spatial breaking symmetry of the initial homogeneous state [1]. In particular, ultrashort laser energy deposition induces local thermal stresses and transient phase changes which modifies materials microstructures and topography [2,3]. The fast interaction with material surfaces results in the formation of anisotropic surface morphology, usually recognized as laser-induced periodic surface structures (LIPSS) or ripples. LIPSS are a special type of arranged nanopatterns, known for their periodicity varying from near laser wavelength down to sub-100 nm scale [4,5]. Their formation generally occurs in a multi-pulse irradiation regime with a fluence below the ablation threshold. Consequently, the first laser pulse modifies the surface topography randomly by creating roughness centers that are perceived by the next pulses. All the consecutive laser pulses impact on a surface being constantly modified by the previous pulses and being roughened constantly, from an initially quasi-flat surface to transiently growing disturbances [6,7,8]. LIPSS can be generated with different periodic scales on the irradiated surface by virtue of the radiative and nonradiative modes of light coupling, which involves polarization effects on the freshly formed roughness centers [9]. Far beyond the diffraction limit, most of the sub-wavelength structures were oriented by the laser polarization, commonly spaced between 50–200 nm in the literature and a priori provoked by local field enhancement on the local roughness [6,10]. They are generally called high spatial frequency LIPSS (HSFL) of type *r* in the literature [11]. The light coupling is composed of scattered waves but also of nonradiative fields that emerge from nanoreliefs and that generate coherent exchanges of optical near fields [9]. Strong enhancement of the oriented electromagnetic field at surface defects trigger local heat confinement that can destabilize the thin laser-induced melt layer. During the sub-surface rarefaction process, periodic nanovoids enclosed under the surface and nanocavities emerge at the surface by cavitation [12]. During the acceleration of the molten surface by the rarefaction wave, a Marangoni-convection instability can be triggered by hydrothermal waves perpendicular to these temperature gradients [13], forming high spatial frequency periodic surface structures.

Furthermore, it was proven that under multi-pulse feedback regimes, there is a strong dependence of the generation of surface roughness and finally periodic nanoripples on the crystallographic surface orientation [2]. More refined nanostructure was obtained on Cr (100) by single-pulse irradiation and a more pronounced LIPSS after two laser pulses as compared to (110) surfaces [14]. The competition between the evolution of transient liquid structures generated in the spallation process and the rapid resolidification of the surface region were proposed to define the types, sizes and dimensions of the nanoscale surface features [14]. Thus, the formation of sub-100nm nanopatterns are clearly dependent on crystallographic orientation and initial surface roughness. Recent works show that it is possible to emancipate from the polarization-dependency by combining two orthogonal linear polarization states. The control of the ultrafast laser pulse polarization, inter-pulse delay and laser fluence leads to the generation of innovative 2D surface morphologies on different scales. Several works focused on the fabrication of 2D-LIPSS in the near-submicron length [15,16,17]. These microstructures can lead to strong modifications on surface wetting [18], tribological and optical properties [19]. Laser-induced textures enable surface functionalities, such as hydrophobicity [20], antibacterial properties [21], coloring [22,23] and wear resistance [24].

Driven by near-field light enhancement, periodic patterns recently approached ultimate scales down to tens of nanometers [25]. A self-organization of unusual arrays of nanocavities of 20 nm diameter with a periodicity down to 60 nm was created on a Nickel surface oriented in (100) plan. Obtained by overcoming the anisotropic polarization response of the surface by a delayed action of cross-polarized laser pulses, this process opens the route for self-arranged topography at the nanoscale highly demanded in advanced technology sectors. Analogous structures can be produced by using other advanced technologies such as electrochemical etching, but they suffer from low throughput and accuracy. Instead, ultrafast laser–surface interactions reveal sub-100 nm self-arranged hexagonal arrays with a high throughout and the highest precision. As the resulting morphology is energy-driven, the purpose of this paper is to explore the formation of unrevealed nanopatterns under regulated energy dose. Beyond the already reported spatial instability triggering hexagonal arrays, we discuss the laser irradiation parameters as well as the material conditions apt to generate a self-organization regime of nanostructuring. We show that a slight modification of the amount and the timescale of the energy delivery perturbs the system and a stable self-organization regime shifts towards another one with new properties. The initial surface roughness features, in particular, the absence of inordinately high peaks and deep valleys is also shown to be a crucial feature to be checked to control the self-organization regime. A large variety of nanostructuring regimes are reported here with an abrupt transition between them. This is a characteristic feature of dissipative structures, including random patterns with concentrated nanoreliefs, 1D nanostripes, and 2D hexagonal arrays. Disordered, labyrinthine and bumpy nanopatterns that arise from a homogeneous metal surface under ultrafast excitation are originally reported.

## 2. Materials and Methods

### 2.1. Material Preparation

A mono-crystalline Nickel (Ni) sample oriented in (100) direction was used during this study. Single crystals are preferred to insure the uniformity of the structuring process. The Ni (100) bar was grown by directional solidification and cut into 10 × 10 × 10 mm^3^ cubes by using a wire saw. Two types of polishing were performed during experiments, the mechanical and electrochemical polishing. The automatic polishing was performed on “Buehler Automet 250” by using a coarse paper of P180, moving successively to P320, P600, P1200 and P2400 followed by a diamond 3 μm and 1 μm and vibratory polishing on “Buehler Vibromet 2” with the colloidal silica 0.05 μm for 17 h, prior to laser irradiations. Electropolishing was performed after automatic polishing on “Struers LectroPol-5” by using stainless steel electrolyte at 25 Volts for 60 s. Both polishing procedures assure mirror-zero scrach samples with an initial arithmetical mean surface roughness (Ra) below 5 nm. The Ra threshold Ra_th_ for nanostructures formation was found to be 5 nm on the Atomic Force Microscopy (AFM), on a scan of 5 × 5 μm. Crystal orientations were checked by X-ray diffraction prior to laser irradiation to ensure the uniform cutting direction.

### 2.2. Laser Setup

A Ti: Sapphire laser from Coherent (Legend Elite Series) was used in this setup. Powered by an integrated Revolution pump laser and seeded by a Vitara-S oscillator, the ultrafast amplifier delivers pulse durations down to 25 fs, a 1 kHz repletion rate, an output power up to 7 W and a λ=800 nm central wavelength. A thin film polarizer at the output of the laser allows us to separate *s* and *p*-polarized components of the beam. A half-wave plate is devoted to tuning the incoming energy on the double-pulse irradiation setup.

To force an isotropic energy deposition on the surface that fosters self-organization free from polarization orientation, a cross-polarized irradiation strategy is followed. The double-pulse irradiation setup consists of a modified Mach–Zehnder interferometer combining the effect of a crossed-polarization with an inter-pulse delay Δt. The aim of this setup is to help to control the laser-induced structure formation process. A first beam splitter divides the incoming laser beam into two beams. These beams take two different optical paths and then encounter a second beam splitter which recombines the two of them. We call the “fixed arm” the optical path of the “reference beam” and “mobile arm” the optical path of the “sample beam”. The length of the mobile arm can be adjusted by the use of a highly accurate motorized stage. From a distance allowing a path shorter to the fixed arm to another one a little bit longer, the optical path difference induces a temporal delay. The “zero delay” Δt=0 corresponds to a spatial and temporal overlap. Temporal overlap is obtained when the two optical paths have the same length and spatial overlap needs a good orientation of the beam splitters. The “zero delay” configuration is obtained when the pattern of interference is the most contrasted. In each arm, we introduce a polarizer in order to obtain the cross-polarization configuration after recombination. An half-wave plate is also introduced in each arm so we get the same energy for the reference and the sample beam, always checked by a power-meter measurement.

After recombination, the laser beam is then collimated by an optical lens with a focal length of 250 mm and irradiates the surface of a sample with a normal incidence. The surface of the sample is set in the focal plane of the lens independently of the thickness of the sample thanks to the sample holder. The size of the laser spot is always measured before irradiation by the D${}^{2}$ method [26] and the spot size of the Gaussian profile at (1/e${}^{2}$) was $2\omega_{0}=56$ μm where $\omega_{0}$ is the beam waist.

### 2.3. Characterization

Surface topography was visualized by using scanning electron microscopy (SEM) of type “Nova NanoSEM” equipped with a field emission gun. The images were taken at 15 kV with Everhart–Thornley detector (ETD), at different magnitudes. Atomic force microscopy (AFM) of type “Bruker Dimension ICON” was used for characterization in 2D and 3D. Images were taken in SCANASYST-AIR imaging mode, by silicon tip on nitride lever, at different scales and at a resolution of 1024 lines per scan. This microscope has a local probe allowing to visualize the surface topography of a sample with a resolution of about 1 nm. Topological investigations and roughness calculation were performed by using NanoScope Analysis software.

## 3. Results

### 3.1. Advanced Surface Topography Control as a Function of Time Delay and Laser Fluence

Femtosecond laser irradiations are performed by controlling several influential parameters. The two main parameters are the laser peak fluence and the time delay between the double pulses. Other substantial parameters are the polarization angle between the two delayed pulses, number of pulses, pulse duration and initial surface roughness. Based on previous experiments, the chosen number of double-pulse sequences ($N_{DPS}$) was 25 pulses [25]. A cross-polarized ultrashort double pulses are applied in order to break the surface isotropy, avoiding the formation of LIPSS. The relative polarization of the two pulses was fixed by an angle of 90°. The chosen pulse duration was 150 fs and the initial arithmetical mean roughness (Ra) was below 5 nm.

**Figure 1:**
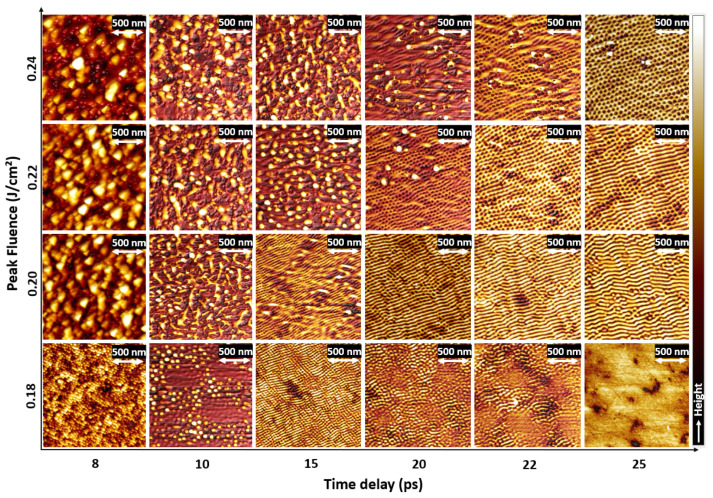
2D AFM images of laser-induced nanopatterns formation on Ni(100) as a function of time delay and peak fluence at a fixed $N_{DPS}$ of 25. The different zones of interest are nanopeaks, nanobumps, nanohumps and nanocavities. They are created progressively at different doses: (0.18 J/cm${}^{2}$; 8 ps), (0.18 J/cm${}^{2}$; 10 ps), (0.18 J/cm${}^{2}$; 15 ps), (0.24 J/cm${}^{2}$; 25 ps).

Figure 1 presents 2D AFM images of laser-induced nanopatterns formation by controlling the laser peak fluence from 0.18 J/cm${}^{2}$ to 0.24 J/cm${}^{2}$ and the time delay (Δt) from 8 ps to 25 ps. Laser parameters are chosen to be below the LIPSS formation threshold for a single pulse, which requires a combined action of the double pulses. At low Δt (8 ps and 10 ps) and at a peak fluence above 0.20 J/cm${}^{2}$, chaotic nanostructures were observed. They are also observed at (15 ps; 0.24 J/cm${}^{2}$) and they are formed for a small time-delay between pulses. Energetically, it is somehow acting similarly to a single pulse process, at a cumulated absorbed fluence higher than the single-pulse absorbed threshold since the small delay leads to a slight decrease of Ni reflectivity with electronic thermal excitation before the second pulse [27]. Up to 15% of reflectivity decrease can be expected for ultrafast photoexcited nickel under electron–phonon nonequilibrium. At 0.18 J/cm${}^{2}$, three different types of nanopatterns were observed at 8 ps, 10 ps and 15 ps. The tallest nanostructures were at Δt = 8 ps and have a shape comparable to a karst peak, so they are named as “nanopeaks”. Nanostructures shape has changed at a larger time delay of 10 ps. Their diameter has increased and their shape was similar to a bump, so they are named as “nanobumps”. We note that the formation of microbumps and nanojets on the metal targets was observed upon single pulse tight focusing ultrashort laser excitation of thin gold films and attributed to Marangoni effect [28]. Our investigation shows that similar structures can be obtained also on irradiated bulks. At Δt = 15 ps, nanostructures height was extensively decreased and they are named as “nanohumps”. Nanostructures were progressively disappeared while increasing the time delay from 15 ps to 25 ps. In this case, the cumulated energy was lower and it can be reasonably assumed that the thermal gradients resulting from local polarization effects were strongly softened by thermal dissipation before the second pulse arrived. The transverse thermal dissipation time due to gradients between two structures at a characteristic distance of 100 nm can be roughly estimated as $\tau_{L}$ = $\frac{\rho C_{i}x^{2}}{2k_{i}}$ where ρ = 7.90 × 10${}^{3}$ kg/m${}^{3}$ is the liquid nickel density, $C_{i}$ = 630 J/kg/K is the heat capacity, $k_{i}$ = 50 W/m/K is the thermal conductivity. Considering a distance of *x* = 50 nm, $\tau_{L}$ ∼ 10 ps. In these conditions, we can estimate that the local polarization memory corresponding to transverse temperature gradients can be rapidly erased by thermal dissipation before the second pulse arrived in the ten of picosecond timescale. At 0.20 and 0.22 J/cm${}^{2}$, we can observe the transition from chaotic structures to stripes, passing by a transition regime at (0.20 J/cm${}^{2}$; 15 ps and 0.22 J/cm${}^{2}$; 20 ps). This transition region has a short-range order, which consists of locally nanobumps and chaotic nanostructures. However, at Δt = 25 ps, labyrinthine patterns were observed [1], which are disordered spatial structures that show a short-range order. At 0.24 J/cm${}^{2}$, we can observe the switch from disordered nanostructures to a pattern of nanocavities when Δt increases, passing a by transition regime from 15 to 20 ps. The hexagonal array of nanocavities were observed previously at similar parameters [25].

### 3.2. Wide Variety of Nanostructure Regimes

**Figure 2:**
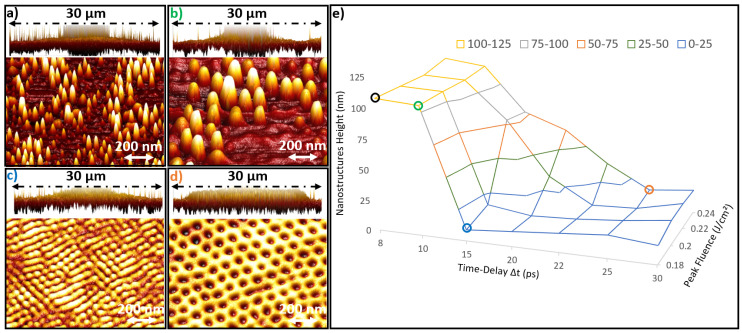
(**a**–**d**) The 3D AFM images of the laser spot topography in the spallation regime and the principal nanostructures types (nanopeaks, nanobumps, nanohumps and nanocavities). (**e**) Maximum nanostructures height as a function of time delay and laser fluence. The colored circles in (**e**) (black, green, blue and orange) present respectively the regions of (**a**–**d**) nanostructures.

Figure 2a–c present 3D AFM images of the principal nanostructures, which correspond to the laser parameters of Figure 1 (a(0.18 J/cm${}^{2}$; 8 ps), b(0.18 J/cm${}^{2}$; 10 ps), c(0.18 J/cm${}^{2}$; 15 ps) and d(0.24 J/cm${}^{2}$; 25 ps)). Figure 2e presents the maximum nanostructure height as a function of time delay and laser fluence. The maximum height was characterized on AFM for each parameter. It was classified into two different categories. One category of nanostructures with a maximum height of almost 110 nm presented in (a) and (b), marked by black and green circles respectively. The other category has a maximum height of almost 20 nm presented in (c) and (d), marked by blue and orange circles respectively. The material surface exhibits a significant swelling suggesting a frustrated ablation regime [29,30].

It can be observed that (a) and (b) have respectively the shape of nanopeaks and nanobumps but with different widths, organization and concentrations. However, (c) and (d) present hexagonal self-organized nanohumps and nanocavities on the surface. Interestingly, (c) exhibits a state with persistent dynamics with oriented stripes independent from the local polarization that may correspond to “spiral defect chaos” [31].

The transition regimes between the two different nanostructures categories of (110 nm and 20 nm) are shown in an almost diagonal way in Figure 1 and Figure 2e. It can be understood that the lost energy by increasing the time delay was covered by the intensification of laser fluence to maintain the instability of the critical phase leading to the presence of these nanostructures. The laser spots images (a, b and c) confirm the occurrence of a spallation regime that exists above the melting threshold, while remaining below phase explosion threshold.

### 3.3. Wide Variety Nanopatterns Morphologies

**Figure 3:**
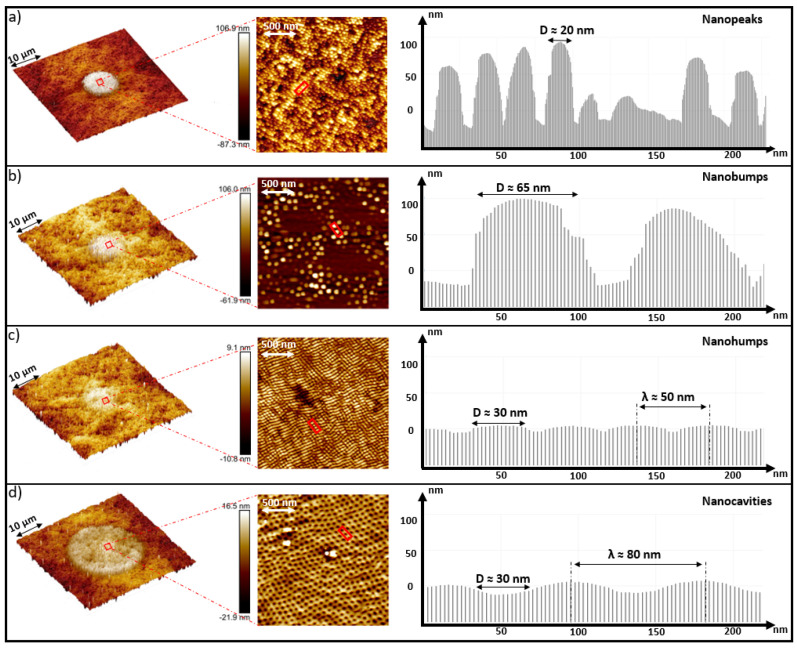
3D AFM images of the principal nanopatterns (**a**–**d**) at different scales, presenting the laser spot region. A scan profile was performed for each type of the principal nanostructures presenting the shape and the periodicity of each type.

Figure 3 presents the 3D AFM images of the principal nanopatterns (a–d) at different scanning scales. (a) presents a nanobump shape comparable to a karst peak. It has a width of ≈20 nm and a height of ≈100 nm and is named a nanopeak. At the same laser peak fluence of 0.18 J/cm${}^{2}$, and just by increasing the time delay from 8 ps to 10 ps, the nanostructures have transformed from (a) to (b). In (b), it has a shape of a nanobump but totally different than in (a). The diameter of the bump is ≈60 nm and the height is ≈100 nm. Moreover, by a slight increase of the time delay from 10 to 15 ps, the nanobump scale was reduced to reach a height less than 10 nm in Figure 3c, which are named nanohumps. Those small hexagonal nanohumps have a periodicity of ≈50 nm. On the other hand, (d) presents self-arranged hexagonal nanocavities with a periodicity of ≈85 nm and a depth of ≈25 nm.

In addition, the laser spot diameter for the nanopatterns (a, b and c) was ≤9 μm. However, the laser spot diameter for the nanocavities shown in (d) was ≤22 μm. Thus, laser fluence is a critical factor in determining the self-organized regime of nanostructures growth.

### 3.4. Nanopatterns Control by Laser Dose

**Figure 4:**
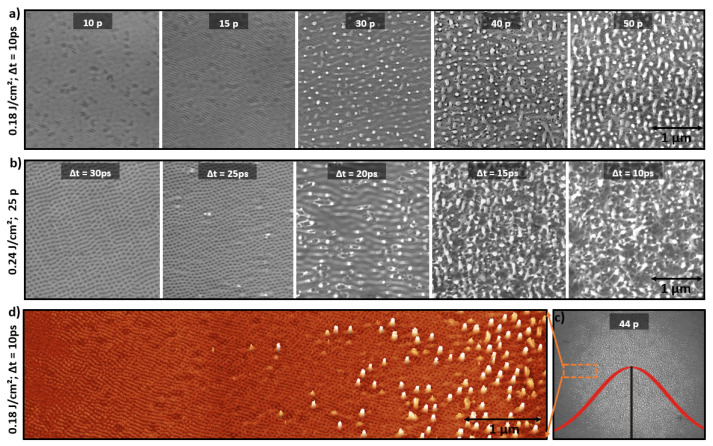
(**a**) 2D scanning electron microscopy (SEM) images presenting the influence of the $N_{DPS}$ in enhancing nanostructures at a fixed time delay. (**b**) 2D SEM images of growing nanopatterns by decreasing the time delay at a fixed peak fluence of 0.24 J/cm${}^{2}$ and $N_{DPS}$ = 25. (**c**) 2D SEM image of the gaussian laser spot at a fixed laser parameters (0.18 J/cm${}^{2}$; 10 ps; $N_{DPS}$ = 44). (**d**) The left region of the gaussian laser spot, showing the significant role of laser fluence in controlling different types of nanostructures.

As presented previously, surface topography and nanopatterns shapes are controlled by laser parameters. Laser dose has a significant role in creating these different patterns. Figure 4a emphasizes the essential role of the $N_{DPS}$ in controlling surface topography and enhancing nanostructures, by fixing the peak fluence to 0.18 J/cm${}^{2}$ and the time delay to 10 ps. Therefore, the surface roughness created by the last pulse is playing an important role in the appearance of different nanopatterns. However, Figure 4b presents SEM images at a fixed $N_{DPS}$ of 25 pulses and a peak fluence of 0.24 J/cm${}^{2}$. As presented in Figure 1, we can observe the transformation from chaotic surface to nanocavities by only inceasing the time delay between the two cross-polarized pulses, passing by a transition regime between 15 and 25 ps. On the other hand, Figure 4c shows the laser spot at the same laser parameters of Figure 4a, but at $N_{DPS}=44$ pulses. Finally, Figure 4d exhibits the left region of the gaussian laser spot of Figure 4c, showing how nanopatterns can be simply regulated by setting a specific laser peak fluence. The presence of different nanostructures on the laser spot periphery stresses the significant role of the laser peak fluence on creating different surface morphologies.

### 3.5. Initial Roughness Effect on Nanostructures Formation

Initial surface topography plays an essential role in nanostructures formation. Several polishing procedures were developed in order to obtain different initial surface topographies, with various initial arithmetic surface roughness (Ra). A Ra > 5 nm, will not permit the formation of different types of previously reported nanohumps and nanocavities.

**Figure 5:**
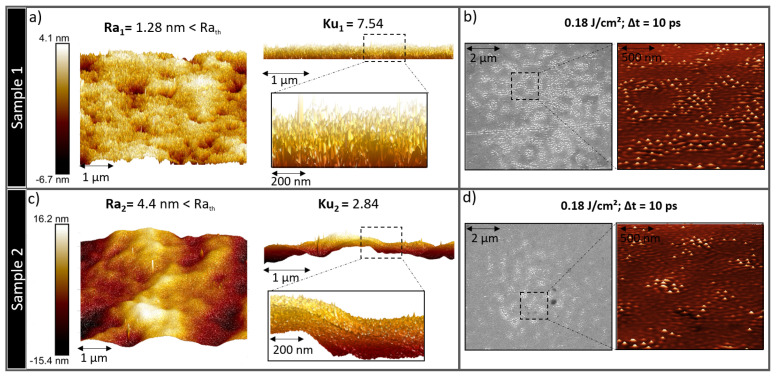
Initial surface topography of two different samples polished by different procedures (Mechanical polishing in (**a**) and Electrochemical in (**c**)). Arithmetic roughness (Ra) and Kurtosis (Ku) were measured and compared for both samples. (**b**,**d**) 2D and 3D SEM images of (**a**,**c**) respectively after laser irradiation. The crucial role of initial type of roughness is observed by comparing the nanostructures concentration in the SEM.

As presented in Figure 5a,c, sample 1 and sample 2 have an initial Ra < Ra_th_. Mechanical polishing was performed for sample 1 by using a coarse paper up to P2400 followed by a diamond polishing of 3 μm and 1 μm and vibratory polishing with colloidal silica of 0.05 μm for several hours, which guarantee a mirror surface with a very low arithmetic roughness. Kurtosis (Ku) statistical parameters was used to characterize the type of initial roughness and sharpness of surface spikes. If Ku > 3 the surface is considered “spiky” and if Ku < 3 the surface is considered “bumpy”. If Ku = 3, the surface has completely random surface roughness. For sample 1, the Ku = 7.54 which is considered spiky surface with a Ra < Ra_th_.

On the other hand, an electrochemical polishing was performed for sample 2 by using stainless steel electrolyte at 25 Volts for 60 s. The surface has an arithmetic roughness Ra = 4.4 nm < Ra_th_ and a Ku of 2.84 which is considered bumpy surface.

By comparing the SEM images of Figure 5b,d, the difference in nanobumps concentration is clearly observed. The spiky surface of sample 1 has a higher surface absorbance compared to the bumpy surface of sample 2, which increased the concentration of nanostructures. The initial type of roughness plays an essential role in controlling surface absorbance and reflectivity. Moreover, AFM characterizations show the difference in the scale of the obtained nanostructures in the two different samples. For sample 1, the maximum nanobumps height was 89 nm and for sample 2 was only 22.3 nm. Thus, the initial surface type of roughness has a prominent role besides the laser parameters, in controlling surface topography at the nanoscale.

## 4. Discussion

Self-organization regimes may result from complex flows conditions depending on competing destabilization and dissipation forces that occur in the transiently laser-induced molten surface. At the considered fluence below ablation threshold, the electrons are excited up to an estimated electronic temperature of 1–2 eV after each laser pulse of the double-pulse sequence. On the electron–phonon relaxation timescale, a thin liquid layer of 10–20 nm thickness is expected to be formed at the surface a few ps after each pulse. During this relaxation stage, the confined temperature gradient of femtosecond laser irradiation induces a pressure wave on the top surface which is followed by a rarefaction wave [12]. Consequently, the melt layer is driven toward the free surface by the rarefaction wave in the same direction of the temperature gradient. This mechanism is vectorially the opposite of the destabilizing effect of the gravitational force acting on a liquid heated from below [32]. In classical Rayleigh–Bénard instability, the gravitational force is directed against the density gradient, pushing down the heavier liquid. In the case of ultrashort laser excitation, the rarefaction below the surface might play a destabilizing role due to the force that results from different pressures in rarefied or non-rarefied liquid, being directed towards the surface and pushing colder liquid inside the warmer upper layer.

Guided by surface tension and rarefaction forces, hydrothermal flows develop a thermoconvective instability at the nanoscale, similar to well-known Rayleigh–Bénard-Marangoni instabilities [33,34]. This mechanism represents a convincing scenario for generating hexagonal nanostructures (nanocavities or nanohumps on the surface) [33]. The Rayleigh–Bénard-like instability occurs since there is a density gradient between the top and the bottom surface, rarefaction wave acts trying to pull the cooler, denser liquid from the bottom to the top [35,36]. Therefore, a Bénard–Marangoni convection occurs along the unstable fluids interface, initiated by small flow perturbations due to recurring surface tension gradients. This Marangoni flow transports thermal energy, that can be characterized by the Marangoni number, comparing the rate at which thermal energy is transported by this flow to the rate at which thermal energy diffuses. Modulated by near field coupling on local roughness, transverse temperature gradients are built parallel to the surface that turns to be unstable [25]. The process starts with a roughness-triggered inhomogeneous energy absorption featured by electromagnetic patterns. They induce a surface thermomechanical fingerprint persisting after electron–ion thermal equilibrium at t=10 ps. Later on, at 15 ps < *t* < 25 ps, the lattice density is strongly influenced by the inhomogeneous shock and rarefaction waves. The surface starts to melt inhomogeneously and the destabilization occurs. The liquid flow is then driven by transverse surface tension forces, rearranging the material from hot spots to colder regions on the surface [13]. Anisotropic temperature gradients generate hydrothermal waves and convection rolls in a disturbed thin liquid layer [37,38]. This generates Marangoni forces balanced by thermal diffusion that reorganize the material over the surface. If isotropic conditions are fulfilled, this can be done in the most compact hexagonal way. Finally, the dimensions of the cell are correlated to the Marangoni number which is equivalent to the ratio between the destabilizing Marangoni force and the viscous restrictive force.

The nature and the orientation of the resulting standing waves are defined by the dimensionless number of Prandtl, which expresses the ratio between the kinematic viscosity and thermal diffusivity and which is leading to hydrothermal waves perpendicular to transverse temperature gradients [37,39]. It makes it possible to understand whether the thermal phenomenon is more or less rapid than the hydrodynamic phenomenon. If the Prandtl number is small, then it should be understood that the phenomenon of thermal conduction is so fast that the velocity profile has little or no effect on the temperature profile. However, for a large Prandtl number, the temperature gradient in the fluid will be strongly influenced by the local velocity of the fluid. It is demonstrated that there exists a critical Prandtl number [40] $P_{r}^{c}=0.25$ such as: For $P_{r}<P_{r}^{c}$, the production of kinetic energy is intensified by isoenergetic redistributions of vorticity that lead to a concentration of the vertical velocity in the vicinity of the center of the cell. Therefore, the convection sets in as a pattern of hexagonal cells with downward motion in the center (*g*-hexagons) which are similar to the obtained nanocavities. On the other hand, for $P_{r}^{c}<P_{r}$, the intensification of kinetic energy production by the expansion of the energy-producing temperature gradient constitutes the reason for the preference of *l* hexagons in high-Prandtl-number convection. Therefore, the conventional hexagonal cells with upward motion in the center (*l*-hexagons) appear at the onset of instability [41,42], which are similar to the obtained nanohumps.

In our case, the time delay plays a major role in controlling the viscosity of the liquid layer. For example, when the time delay is higher, the second pulse shock wave reacts with a surface that already was cooling down, promoting the formation of nanohumps at low irradiation fluence. The mechanism ends up with resolidification of the liquid fluid, when the surface lattice is cooling and the Marangoni forces decrease (after *t* = 100 ps) [13].

On the other hand, it was observed that the cavitation-induced roughness is likely to play a key role in triggering the generation of high-frequency LIPSS upon irradiation by multiple laser pulses. Large-scale molecular dynamics simulation demonstrated that the competition between the evolution of transient liquid structures generated in the spallation process and the rapid resolidification of the surface region are defining the types, sizes and dimensions of the nanoscale surface features [14]. Moreover, associated to void formation and growth, liquid filaments with a high aspect-ratio perpendicular to the surface were reported when the spallation process starts in atomistic computations targeting the nanohydrodynamics phenomena [43]. The observed nanospikes on the previous study closely resemble the obtained nanopeaks in our study. To go further ahead in this analysis, complementary microstructural investigations are needed to enlarge the study of the physical mechanisms leading to the formation of the observed nanopatterns.

## 5. Conclusions

Various nanopatterns corresponding to distinct self-organization regimes induced by convection mechanism at the nanoscale were investigated on femtosecond laser-irradiated nickel surfaces. Forming far from equilibrium, complex patterns of structures are revealed caused by intensive light energy exchange with a dissipative environment. An hydrothermal mechanism is highlighted by the appearance of two competing nanostructures, nanohumps and hexagonal nanocavities. The nanorelief is characterized by mounds and depressions where the unbalance and the respective predominance is shown to be tunable by the time-delay and the fluence. Observed for (0.18 J/cm${}^{2}$; Δt=15 ps) and (0.24 J/cm${}^{2}$; Δt=25 ps) both kind of nanostructures have 30 nm diameter and tens of nanometers height. Nanopeaks and nanobumps were observed at (0.18 J/cm${}^{2}$; Δt=8 ps) and (0.18 J/cm${}^{2}$; Δt=10 ps). The nanopeaks have 20 nm width and 110 nm height whereas the nanobumps exhibit a 65 nm width and 100 nm height. Potentially resulting from thermo-convective instabilities, they were formed during the competition of the pressure-gradient (via surface tension and cavitation) forces and the rapid resolidification of the molten surface region. Successively implying emergence, growth, amplification and regulation of the pattern formation, laser parameters as peak fluence, time delay, and number of double-pulses sequences play a significant role on modifying surface morphology and topography. Furthermore, the initial surface roughness and type of roughness is another essential feature to be controlled to switch from a regime of self-organization to another one. The involved instabilities being expected in most of ultrafast-laser irradiated materials, the process reported in this paper is a priori generalizable to any metals in order to design a predefined surface morphology at the nanoscale. The production of these nanostructures can enable unique surface functionalizations toward the control of mechanical, biomedical, optical, or chemical surface properties on a nanometric scale [44].

## Data Availability

Not applicable.

## Abbreviations

The following abbreviations are used in this manuscript:

LIPSS	Laser-Induced Periodic Surface Structures
HSFL	High Spatial Frequency LIPSS
SEM	Scanning Electron Microscopy
AFM	Atomic Force Microscopy
Ra	Arithmetic Roughness
Ra_th_	Arithmetic Roughness Threshold
Ku	Kurtosis
$P_{r}$	Prandtl Number
$P_{r}^{c}$	Critical Prandtl Number
$N_{DPS}$	Number of Double-Pulses Sequences
Δt	Time-Delay Between the Double-Pulses
